# The Two Endocytic Pathways Mediated by the Carbohydrate Recognition Domain and Regulated by the Collagen-like Domain of Galectin-3 in Vascular Endothelial Cells

**DOI:** 10.1371/journal.pone.0052430

**Published:** 2012-12-26

**Authors:** Xiaoge Gao, Dan Liu, Yuying Fan, Xinzhi Li, Huiting Xue, Yingyun Ma, Yifa Zhou, Guihua Tai

**Affiliations:** 1 School of Life Sciences, Northeast Normal University, Changchun, PR China; Institute of Molecular and Cell Biology, Singapore

## Abstract

Galectin-3 plays an important role in endothelial morphogenesis and angiogenesis. We investigated the endocytosis of galectin-3 in human vascular endothelial cells and showed that galectin-3 could associate with and internalized into the cells in a carbohydrate-dependent manner. Our work also revealed that galectin-3 was transported to the early/recycling endosomes and then partitioned into two routes – recycling back to the plasma membrane or targeting to the late endosomes/lysosomes. Various N- and C-terminal truncated forms of galectin-3 were constructed and compared with the full-length protein. These comparisons showed that the carbohydrate-recognition domain of galectin-3 was required for galectin-3 binding and endocytosis. The N-terminal half of the protein, which comprises the N-terminal leader domain and the collagen-like internal repeating domain, could not mediate binding and endocytosis alone. The collagen-like domain, although it was largely irrelevant to galectin-3 trafficking to the early/recycling endosomes, was required for targeting galectin-3 to the late endosomes/lysosomes. In contrast, the leader domain was irrelevant to both binding and intracellular trafficking. The data presented in this study correlate well with different cellular behaviors induced by the full-length and the truncated galectin-3 and provide an alternative way of understanding its angiogenic mechanisms.

## Introduction

Galectin-3 (Gal-3) is a member of the galectin family, which is characterized by a conserved sequence within the carbohydrate recognition domain (CRD) that has an affinity for β–galactoside residues [1]–[3]. Gal-3 is a 30 kD protein with distinct N- and C-terminal structural domains. The C-terminal half (i.e., the CRD), composed of approximately 130 amino acids that form a globular structure, accommodates the entire carbohydrate-binding site and is thus responsible for the lectin activity of Gal-3. The N-terminal half (referred to as NT) contains 110–130 amino acids and is highly conserved among the Gal-3 molecules that have been isolated from different species [2], [4]. Self-association of Gal-3 is mostly dependent on the NT [5]. Different parts of the NT have been sub-classified into a short N-terminal leader domain (LD), which corresponds to the first 12 amino acids, and a collagen-like internal repeating domain (RD), which contains 7–14 tandem repeats of short amino acid segments (Pro–Gly–Ala–Tyr–Pro–Gly) [6], [7].

Like other members of the family, Gal-3 lacks a signal peptide but can be secreted via a non-classical transport pathway [8]. Extracellular Gal-3, via binding with its glycol-ligands on the cell surface or in the extracellular matrix, participates in multiple biological processes including tumor angiogenesis and metastasis [9]–[15]. Early studies have shown that Gal-3 can bind to human umbilical vein endothelial cells (HUVECs) and induce their migration in vitro. Gal-3 has also been shown to induce new blood vessel formation in the matrigel and tumor anginogenesis in vivo [14], [16]–[18]. Lactose and modified citrus pectin, a competitive disaccharide and polysaccharide, respectively, inhibit these events, suggesting the involvement of carbohydrate recognition by the CRD [19]. Gal-3 loses its angiogenic activities when its NT is cleaved at amino acid 108 by collagenase digestion, suggesting that the self-association conferred by the NT is also involved [17]. Markowska et al. investigated the mechanism of Gal-3-induced angiogenesis and demonstrated that Gal-3 acts as a mediator in both vascular endothelial growth factor (VEGF) and basic fibroblast growth factor (bFGF)-mediated angiogenic response [16]. Recently, it has been shown that Gal-3 modulates cell surface expression and activation of vascular endothelial growth factor receptor 2 (VEGF-R2) in human endothelial cells [18]. In addition to promoting angiogenesis, it is well established that Gal-3 mediates metastatic cell adhesion to the endothelium [20]. The concentration of circulating Gal-3 increases in the sera of some cancer patients [21]. An increase in circulating Gal-3 has been shown to increase the adhesion of metastatic cells to endothelial cells [22].

How Gal-3 interacts with endothelial cells has been the subject of much research because it is fundamental to understanding Gal-3-mediated angiogenesis and hematogenous metastasis. Until now, most studies have focused on cell surface interactions [11], [16], [18], and there have been no reports concerning the endocytosis of Gal-3 into endothelial cells. Because endocytosis affects the cellular distribution and turnover rate of cell surface receptors, it is conceivable that the endocytosis of Gal-3 would closely relate to its function.

Although the endocytosis of Gal-3 has been studied in several other cell types, its endocytic pathways and mechanisms are still poorly understood. Earlier studies by Zhu and Ochieng et al showed that Gal-3 can be rapidly internalized by breast carcinoma cells [23], [24]. This process was inhibited by filipin but not chlorpromazine. Schneider et al. studied the apical uptake of Gal-3 in polarized MDCK cells. They found that exogenous Gal-3 was rapidly taken up and that the internalized Gal-3 displayed colocalization with Rab11, a marker for recycling endosomes [25]. Recently, a comparative study of the endocytosis of Gal-3 in different macrophage-like cell types was published. This study demonstrated that Gal-3 endocytosis in M2 cells is carbohydrate dependent, while in M1 cells it is carbohydrate independent. Besides, Gal-3 has been shown to interact directly with membrane lipids and spontaneously penetrate the lipid bilayer of lipsomes in either direction [26].

In this paper, we studied the binding, endocytosis and intracellular trafficking of full-length and N- and C-terminal-truncated recombinant human Gal-3 in human vascular endothelial cells HUVEC and HMEC-1 (human microvecular endothelial cells). Our results showed that endocytosed Gal-3 followed two intracellular transport pathways – either recycling to the plasma membrane or targeting to the late endosomes/lysosomes. The CRD and RD played distinct roles in Gal-3 binding, endocytosis and pathway selection.

## Results

### Preparation of the Full-length and Truncated form of Gal-3

**Figure 1:**
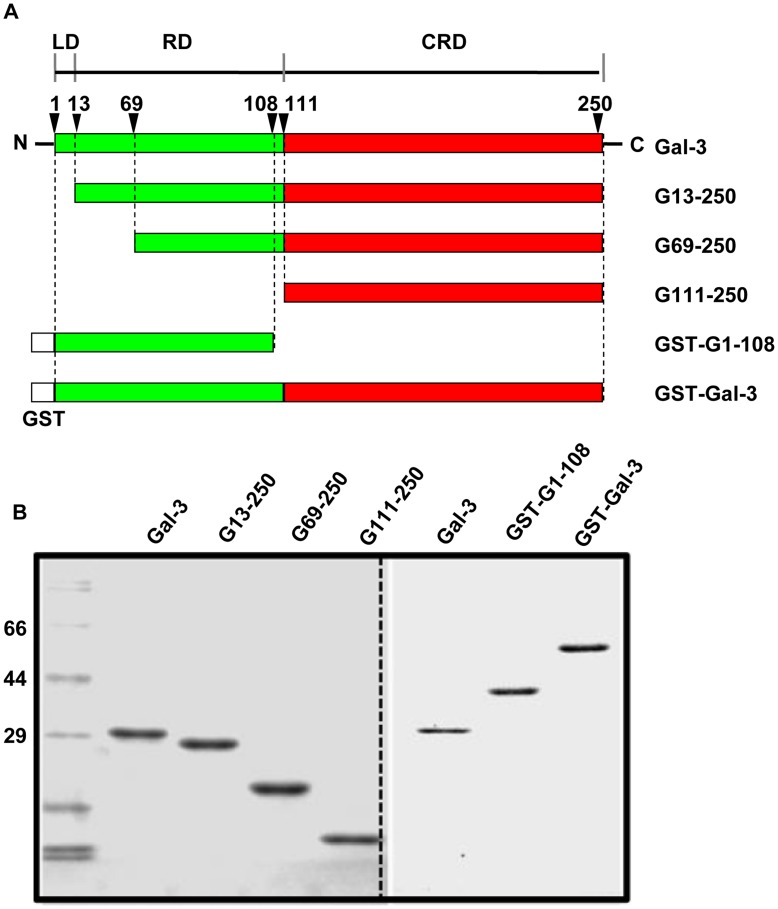
Preparation of the full-length and truncated Gal-3. A: Schematics of the full-length and truncated Gal-3. B: Purity was confirmed by 12% SDS-PAGE. Gal-3 refers to the full-length protein, composed of 250 amino acid residues; G13-250, G69-250 and G111-250 refer to the N-terminal truncated forms of Gal-3, containing the amino acids from 13 to 250, 69 to 250, and 111 to 250 respectively; GST-Gal-3 refers to the full-length Gal-3 fused to the GST tag; GST-G1-108 refers to the N-terminal 108 amino acids fused to the GST tag.

Human Gal-3 is composed of 250 amino acids, with the NT comprising approximately 110 amino acids at the N-terminus and the CRD approximately 130 amino acids at the C-terminus. The NT contains a short LD (the first 12 amino acids) followed by a collagen-like RD [1], [2], [17]. It has been shown that the NT can mediate self-association and confers the cross-linking properties of Gal-3; the CRD can bind glycol-ligands and confers the lectin activities of Gal-3; the LD governs Gal-3 secretion and nuclear localization; and the RD harbors the cleavage sites for collagenases and matrix metalloproteinases and is relevant to tumor growth and angiogenisis [2], [17], [19]. To study Gal-3 endocytosis and the roles of the different structural domains, six constructs were cloned, as shown in Figure 1A. The proteins were affinity purified either on Lactose-Sepharose CL-6B (for Gal-3, G13-250, G69-250, and G111-250) or on Glutathione Sepharose 4B (for GST-G1-108 and GST-Gal-3) and examined by SDS-PAGE (Figure 1B). For microscopic studies, a portion of these proteins were labeled with DTAF. The labeling intensity was evaluated by the ratio of absorbance at 490 nm to 280 nm, which showed that all the proteins had equivalent labeling intensity (data not shown).

### Exogenous Gal-3 Specifically Associated with HUVECs in a Concentration- and Time- Dependent Manner

**Figure 2:**
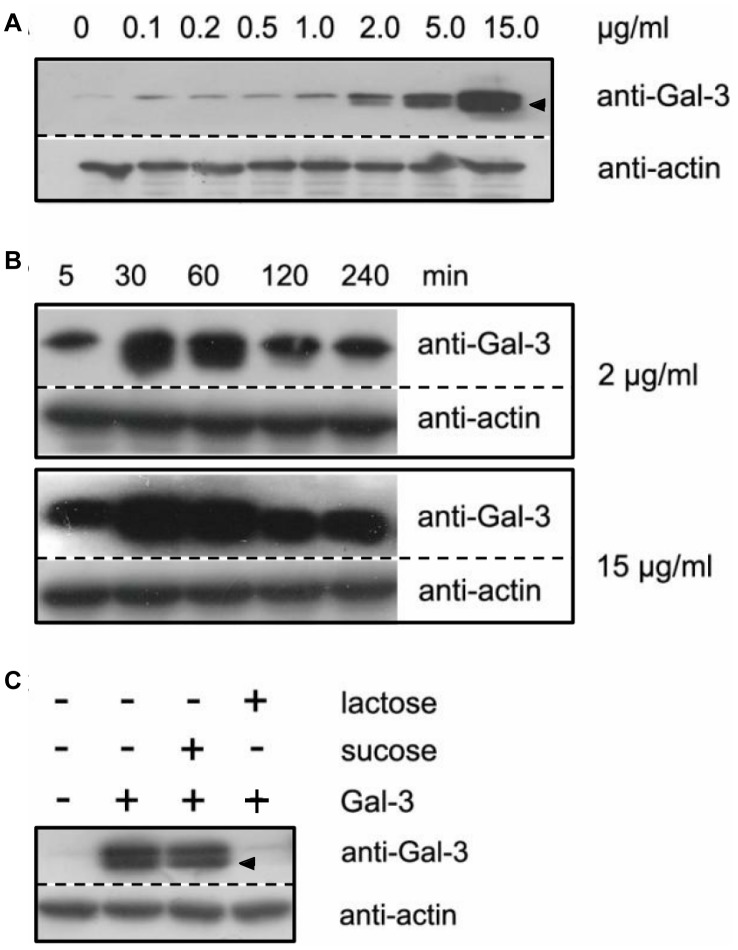
The association of Gal-3 with HUVECs. A: Cell association is concentration dependent. The cells were incubated for 30 min in SFM containing 0, 0.1, 0.2, 0.5, 1, 2, 5 or 15 µg/ml Gal-3. B: Cell association is time dependent. The cells were incubated in SFM containing either 2 or 15 µg/ml Gal-3 for 5, 30, 60, 120, or 240 min. C: Cell association is lectin specific. The cells were incubated for 30 min in SFM containing 15 µg/ml Gal-3 in the presence or absence of 50 mM lactose or sucrose. After incubation, the cells were extensively washed with cold SFM. Western blotting analysis was performed with anti-Gal-3 and anti-actin antibodies. Arrowhead, degraded form of Gal-3.

The HUVECs were incubated with 0, 0.1, 0.2, 0.5, 1, 2, 5 and 15 µg/ml of full length Gal-3 at 37°C for 30 min. Non-binding material was then washed off and the cells were analyzed by western blotting for Gal-3 with actin as a loading control (Figure 2A). Without exogenous Gal-3 (0 µg/ml), the intensity of Gal-3, i.e., endogenously expressed Gal-3, was very weak under the conditions used. In the presence of the exogenous Gal-3, the intensity increased slightly at 0.1, 0.2 and 0.5 µg/ml and dramatically at ≥1 µg/ml. Thus, in our experiments, exogenous Gal-3 was able to associate with the cells, and the level of association was proportional to the concentration of Gal-3 in the medium. A band that migrated faster than the intact Gal-3 (arrowhead, Figure 2) was frequently noticed. This band might be a degraded form of Gal-3 that was generated by the proteolytic enzymes in the culture. Gal-3 hydrolysis in culture has been frequently reported [27].

The time course of the association was investigated by incubating the cells with either 2 or 15 µg/ml Gal-3 for 5, 30, 60, 120, and 240 min. As shown in Figure 2B, a significant amount of Gal-3 was already associated with the cells after the 5 min incubation. Association increased rapidly and reached a maximum at approximately 30–60 min. Then, association began to decrease and leveled off at 120 and 240 min. Thus, Gal-3 association with cells was time dependent. This result suggested that there might be a rapid endocytosis step that is followed by a rapid exocytosis step and that then equilibrium is reached between endocytosis and exocytosis. It is unlikely that protein degradation was the cause for these shifts in concentration because the amount of Gal-3 remained constant between 120 and 240 min instead of decreasing continuously.

To investigate if the association of Gal-3 was a specific event, cells were incubated with Gal-3 in the presence of lactose or sucrose. Lactose is a disaccharide that has been previously shown to compete with natural ligand (s) recognition by Gal-3, and sucrose is a non-competitive disaccharide. As shown in Figure 2C, Gal-3 cell association was completely lost in the presence of lactose, whereas the amount of association did not change in the presence of sucrose. These data indicate that the association was a specific event that involved carbohydrate recognition.

### The Exogenous Gal-3 was Endocytosed

**Figure 3:**
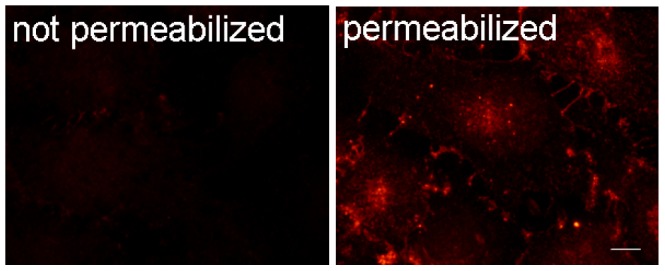
The endocytosis of Gal-3. The HUVECs were incubated with biotinylated Gal-3 for 30 min and then either permeabilized or not before examination by IF analysis with Rhodamine- streptavidin. Images from a fluorescent microscope. Scale bar, 10 µm.

To determine if cell-associated Gal-3 was endocytosed, cells were incubated with biotinylated Gal-3 at 37°C for 30 min. Cells were either permeabilized or not before examination by IF with Rhodamine-streptavidin. As shown in Figure 3, there was intense fluorescence from permeabilized cells but little from non-permeabilized cells. This indicated that exogenous Gal-3 was endocytosed and that, under our experimental conditions, the vast majority of cell-associated Gal-3 was inside the cells rather than at the cell surface.

### Gal-3 was Transported to the Late Endosomes/lysosomes (LE/lysosomes) via the Early/recycling endosomes (EE/RE)

**Figure 4:**
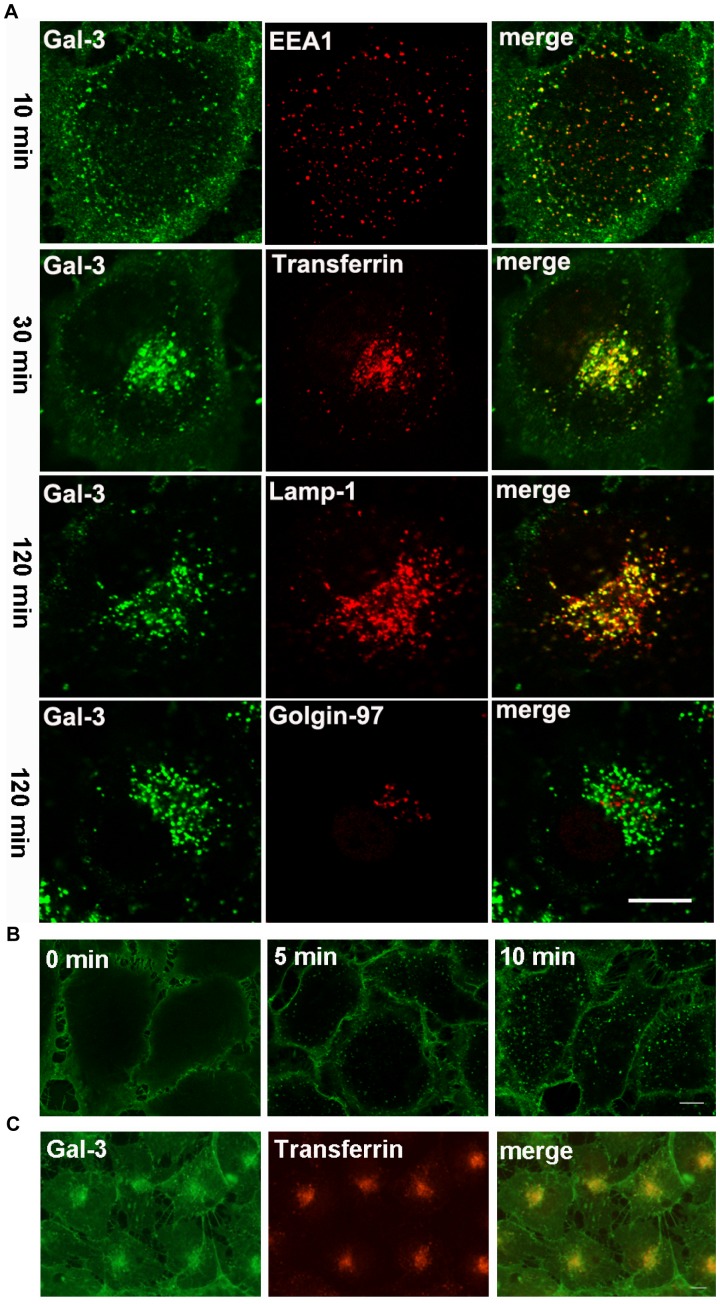
The endocytic pathways of Gal-3 in HUVEC. A: The cells were incubated with DTAF-Gal-3 and Cy3-transferrin (only at 30 min) at 37°C for 10, 30, or 120 min and were then processed for IF analysis with antibodies against EEA1, Lamp-1 and Golgin-97. B: The cells were incubated with DTAF-Gal-3 at 4°C for 60 min. After washing, the cells were either fixed immediately (0 min) or transferred to 37°C for 5 min or 10 min and then fixed. C: The cells were incubated with DTAF-Gal-3 and Cy3-transferrin at 20°C for 60 min. The images were obtained with a confocal microscope (A and B) or a fluorescence microscope (C). Scale bar, 10 µm.

To study the kinetics of Gal-3 endocytosis, DTAF-labeled Gal-3 (DTAF-Gal-3) was incubated with the HUVECs at 37°C for various times and examined under a confocal microscope (Figure 4A). At approximately 10 min, Gal-3 appeared as vesicles scattered in the cytoplasm and co-localized with the EE marker EEA1. At approximately 30 min, the vesicles accumulated in the juxtanuclear region, reminiscent of the RE, and co-localized well with the co-internalized transferrin. This compact pattern gradually disappeared, and the remaining vesicles re-organized into a loose pattern reminiscent of the LE. At 120 min, more than 80% of Gal-3 containing vesicles co-localized with the LE/lysosomes marker Lamp-1. The co-localization remained for more than 10 h (data not shown). There was no co-localization at any time with Golgin-97, a marker for the trans-Golgi and TGN. Together, these data indicated that Gal-3, or a fraction of it, was transported to the LE/lysosomes via the EE/RE.

### Gal-3 Endocytosis and Intracellular Trafficking were Inhibited at Low Temperatures

To study if the endocytosis of Gal-3 was blocked at low temperatures, endocytosis was induced at 4°C and 20°C (Figure 4B, 4C). After incubation at 4°C for 60 min (Figure 4B, 0 min), fluorescence was observed only on the cell surface. There were no vesicles in the cytoplasm. Thus, Gal-3 was bound to the cell surface, and its endocytosis was blocked at 4°C. However, the endocytosis quickly resumed upon transferring cells to 37°C (temperature release). Small vesicles near the PM were clearly seen after 5 min of temperature release (Figure 4B, 5 min), and larger vesicles were observed all over the cytoplasm after 10 min (Figure 4B, 10 min). After incubation at 20°C for 60 min, Gal-3 was endocytosed and showed both accumulation at the juxtanuclear region and co-localization with the co-internalized transferrin (Figure 4C). No colocalization with Lamp-1 was observed after 4 h (data not shown). Thus, the endocytosis of Gal-3 to the EE/RE was not blocked at 20°C, but its further transport to the LE/lysosomes was inhibited.

**Figure 5:**
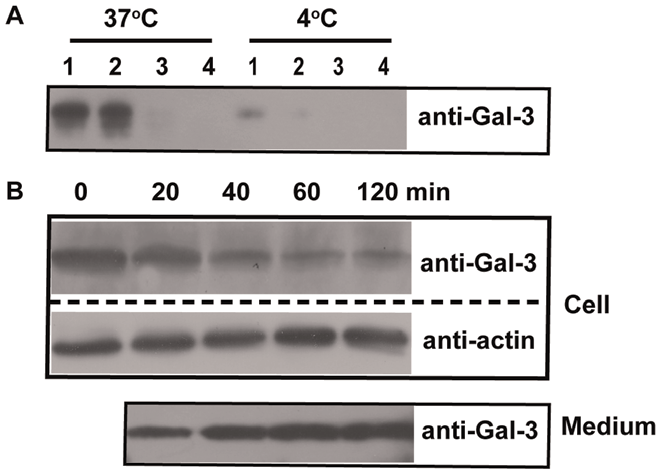
The exocytosis of Gal-3. A: HUVECs were incubated in SFM with 15 µg/ml Gal-3 for 30 min at 37°C and then washed five times at 4°C. The cells were placed in fresh SFM and then incubated at 37°C or 4°C for a total of 120 min with fresh media changes every 30 min. The media collected from each time point were precipitated by TCA and analyzed by western blotting. B: The HUVECs were incubated with 15 µg/ml Gal-3 for 30 min at 4°C and then washed five times at 4°C. The cells were then placed in fresh SFM and incubated at 37°C for 0, 20, 40, 60, or 120 min. Both the cells and the media were analyzed with western blotting.

**Figure 10:**
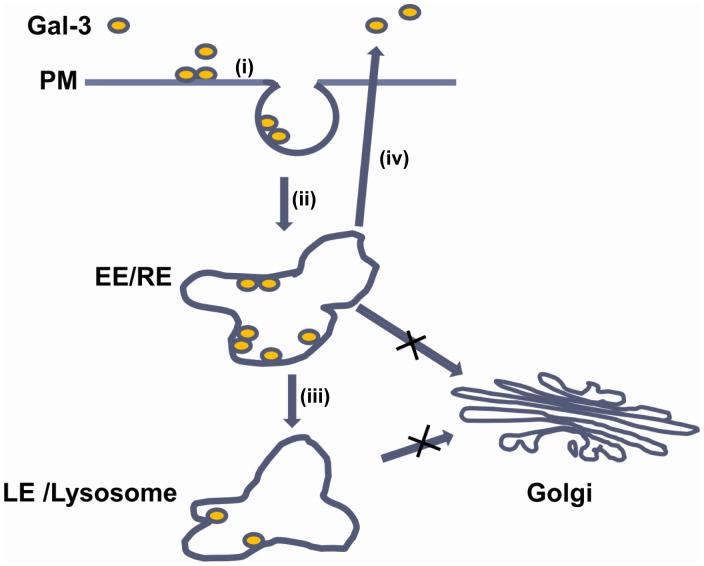
Schematic of the endocytic pathways of Gal-3 in endothelial cells. (i) Gal-3 bound with the glycol-ligand on the plasma membrane (PM); (ii) Gal-3 was endocytosed and transported to the early/recycling endosomes (EE/RE); (iii) A fraction of the endocytic Gal-3 was recycled to the PM and then released into the medium; (vi) A fraction of the endocytic Gal-3 was transported to late endosome/lysosomes (LE/lysosomes).

### A Fraction of Gal-3 was Exocytosed via the EE/RE

Our time course experiment (Figure 2B) suggested that some endocytosed Gal-3 might be exocytosed. To test this possibility, HUVECs were incubated with Gal-3 for 30 min at 37°C. Under these conditions, cell-associated Gal-3 was mainly located inside the cells (Figure 3). The cells were then washed, placed in fresh medium and incubated at either 37°C or 4°C for a total of 120 min, with fresh medium changes every 30 min. The medium that was collected at each time point was examined by western blotting. As shown in Figure 5A, a significant amount of Gal-3 was detected in the first two 30 min incubations at 37°C, some in the third and none in the fourth. Comparatively, only a small amount of Gal-3 was detected in the first 30 min at 4°C. These data indicated that some endocytosed Gal-3 was indeed released into the medium and that most was released within the first 60 min. In addition, exocytosis was inhibited at 4°C.

To quantitatively measure Gal-3 exocytosis, five sets of cells were identically treated with Gal-3 at 4°C to synchronize Gal-3 at the PM as indicated in Figure 4B. After washing off non-binding Gal-3, each set of cells was placed in fresh medium and incubated at 37°C for 0, 20, 40, 60 or 120 min, respectively. At each time point, both the cells and the medium were analyzed by western blotting. As shown in Figure 5B, the amount of Gal-3 in the cells gradually decreased until 60 min, but changed little from 60 to 120 min. Consistent with this trend, the amount of Gal-3 in the medium gradually increased until 60 min and remained largely unchanged from 60 to 120 min. It can be estimated from these data that, at steady state, approximately 70% of cell-associated Gal-3 was exocytosed and that the majority was exocytosed in less than 60 min. The quick rate of exocytosis in both experimental conditions (Figure 5A and 5B) suggested that exocytosis mainly occurred at the EE/RE rather than at the LE/lysosomes, because morphological analysis showed that Gal-3 was mainly observed at the EE/RE and that the majority of Gal-3 did not arrive at the LE/lysosomes in less than 60 min (Figure 4A).

### The Endocytosis of Gal-3 was Dependent on the Presence of the CRD

**Figure 6:**
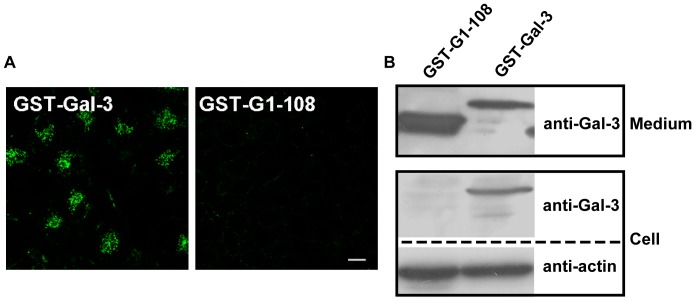
Gal-3 did not associate with HUVECs in the absence of the CRD. A: The HUVECs were incubated with 15 µg/ml DTAF-GST-Gal-3 or DTAF-GST-G1-108 at 37°C for 120 min and examined with a confocal microscope. Scale bar, 10 µm. B: The HUVECs were incubated with 15 µg/ml GST-Gal-3 or 30 µg/ml GST-G1-108 at 37°C for 30 min. The amount of proteins added to the medium is shown in the top panel (Medium). The cells were extensively washed and analyzed by western blotting with antibodies to Gal-3 and actin.

Gal-3 consists of a CRD and an NT. The CRD has been implicated in carbohydrate recognition and the NT in protein-protein interactions. Both the CRD and NT have been shown to mediate the endocytosis of Gal-3 in macrophage-like cells [26]. To study the potential roles of the CRD and NT in Gal-3 endocytosis in HUVECs, the entire CRD was deleted. The remaining NT was fused to GST to produce a CRD-null construct, GST-G1-108 (Figure 1). The ability of GST-G1-108 to associate with and internalized into cells was examined and compared to that of the full-length fusion protein GST-Gal-3. As shown by morphological analysis (Figure 6A), cells treated with DTAF-labeled GST-Gal-3 displayed very bright fluorescence compared to cells treated with DTAF-labeled GST-G1-108. Cells treated with GST-G1-108 had very weak fluorescence that was negligible compared to that in the full-length construct. As shown by western blotting (Figure 6B), GST-Gal-3 associated well with cells. In contrast, GST-G1-108 did not associate with the cells even at double the concentration of the full-length construct (compare the intensities of GST-G1-108 and GST-Gal-3 in the “Medium” panel). Thus, both morphological and western blotting analysis indicated that the NT alone could not mediate endocytosis and that the presence of the CRD was absolutely required. This result was consistent with our observation that cell association was inhibited by lactose (Figure 2C).

### Gal-3 N-terminal Domains Played Regulatory Roles during Gal-3 Endocytosis

**Figure 7:**
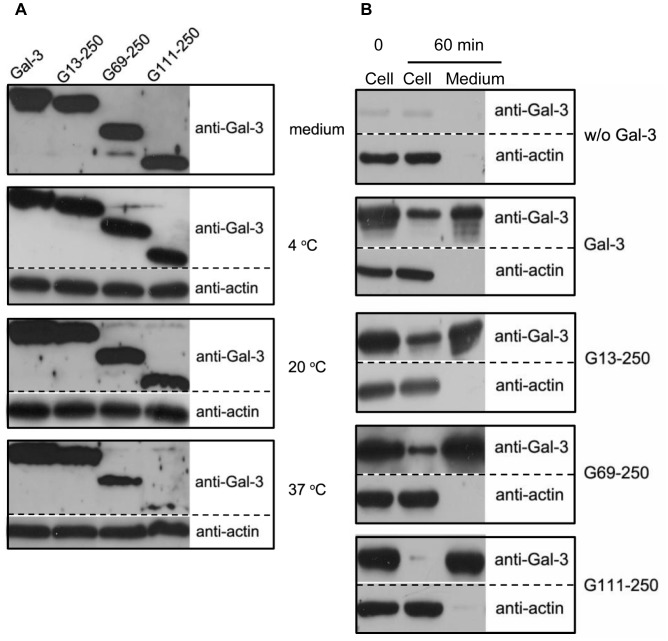
The roles of Gal-3 N-terminal domains in endocytosis and exocytosis. A: The association of various Gal-3 proteins with HUVEC at different temperatures. The cells were incubated with 15 µg/ml Gal-3, G13-250, G69-250 or G111-250 for 60 min at 4°C, 20°C, or 37°C. The amount of protein added in the medium is shown in the top panel (medium). Cell associated proteins were analyzed by western blotting with polyclonal antibodies against Gal-3 and actin. B: The exocytosis of various Gal-3 proteins by the cells. The cells were incubated with or without (w/o) Gal-3, G13-250, G69-250 and G111-250 at 4°C for 30 min. After extensive washing, the cells were either lysed immediately (0 min) or incubated in fresh SFM at 37°C for 60 min. After incubation, both the media and the cells were analyzed with western blotting.

To investigate potential roles for the N-terminal domains of Gal-3 during endocytosis, three N-terminal-truncated forms of Gal-3, G13-250, G69-250 and G111-250, were prepared (Figure 1). G13-250 lacked the LD, G69-250 was missing the LD and part of the RD, and G111-250 lacked both the LD and the entire RD. The ability of each construct to associate with HUVEC was compared at 4°C, 20°C and 37°C (Figure 7A). Here, a polyclonal antibody against Gal-3 was used for the western blot analysis because previous research has shown that the epitope for the monoclonal antibody A3A12 is in the NT, which would render G69-250 and G111-250 unrecognizable by the A3A12 antibody. As shown in the top panel (Medium) of Figure 7A, the polyclonal antibody recognized Gal-3, G13-250, G69-250 and G111-250 with equivalent sensitivities. At both 4°C and 20°C, there were no significant differences between these constructs. At 37°C, however, their intensities were markedly different. The intensity of G13-250 was similar to that of the full-length Gal-3, G69-250 had a significantly lower intensity than G13-250, and the intensity of G111-250 was significantly lower than that of G69-250. According to our data shown above, full-length Gal-3 bound to the PM at 4°C, internalized to the EE/RE at 20°C, and was transported to the LE/lysosomes or exocytosed at 37°C. Thus, the N-terminal domains have little effect on Gal-3 binding and endocytosis to the EE/RE, but dramatically influence later transport. To quantify the proportion of proteins that were exocytosed or sent to the LE/lysosomes, the constructs were synchronized at the cell surface and then allowed to internalize and externalize at 37°C for 1 h. As shown in Figure 7B, approximately 70% of Gal-3, 70% of G13-250, 85% of G69-250 and more than 95% of G111-250 was exocytosed, while 30% of Gal-3, 30% of G13-250, 15% of G69-250 and less than 5% of G111-250 remained in the cells. The remaining portion of Gal-3 construct was mainly transported to the LE/lysosomes (Figure 4A & Figure S1). These data indicated that the N-terminal domains played a pivotal role as to which route Gal-3 would follow, either recycling or transport to the LE/lysosomes. Deleting the LD (i.e., G13-250) had little effect; deleting the LD plus part of the RD (i.e., G69-250) significantly inhibited the route involving the LE/lysosomes; and deleting the LD plus the entire RD (G111-250) almost completely blocked the route involving the LE/lysosomes. Thus, the N-terminal domains, and especially the RD, were required for sorting the full-length protein toward transport to the LE/lysosomes. Without the RD, nearly all protein molecules were diverted into the recycling route.

**Figure 8:**
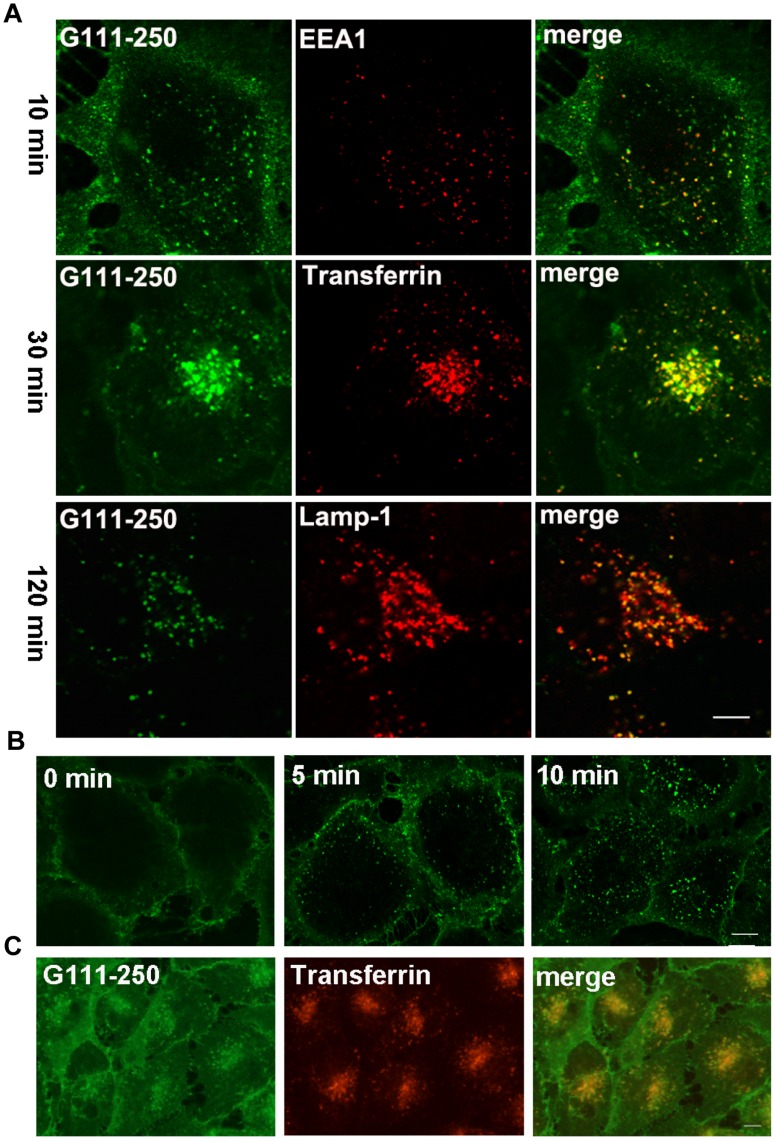
The endocytic pathways of G111-250 in HUVEC. A: The kinetics of G111-250 endocytosis. DTAF-G111-250 was incubated with HUVEC at 37°C for 10 min, 30 min, and 120 min and then processed for IF analysis as described in Figure 4. B: The endocytosis of G111-250 was inhibited at 4°C and rapidly resumed upon return to 37°C. C: The intracellular trafficking of G111-250 was inhibited at 20°C. The experimental procedures for B and C were identical to those in Figure 4B, and 4C, respectively. Scale bar, 10 µm.

The potential endocytic roles of different Gal-3 N-terminal domains were also investigated using morphological methods with DTAF-labeled proteins. Data from the construct G111-250 are presented in Figure 8. G111-250 was the shortest Gal-3 fragment and differed the most from the full-length construct in terms of protein structure and endocytic pathway. When endocytosed at 37°C (Figure 8A), G111-250 displayed co-localization with EEA1 at 10 min and with transferrin at 30 min, similar to the full-length Gal-3. However, it had very weak fluorescence at 120 min, which was drastically different from the full-length Gal-3. Interestingly, the weak fluorescence of G111-250 also co-localized with Lamp1. The early stages of G111-250 transport were confirmed by low-temperature inhibition assays. After incubation at 4°C for 60 min (Figure 8B), G111-250 was observed at the PM, but rapidly internalized upon temperature increase to 37°C. After incubation at 20°C for 60 min, G111-250 accumulated at the EE/RE as indicated by its co-localization with transferrin (Figure 8C). Together, these data indicated that the endocytosis of G111-250 from the plasma membrane to the EE/RE was similar to that of the full-length construct, but only a very small proportion reached the LE/lysosomes. G69-250 behaved similarly, but a higher proportion of G69-250 reached the LE/lysosomes (Figure S1). There were no significant differences between G13-250 and the full-length construct (Figure S1). Taken together, the morphological data confirmed that Gal-3 N-terminal domains had little effect on Gal-3 binding and endocytosis to the EE/RE, but that they significantly affected further transport.

### The Two Transport Pathways in HMEC-1

**Figure 9:**
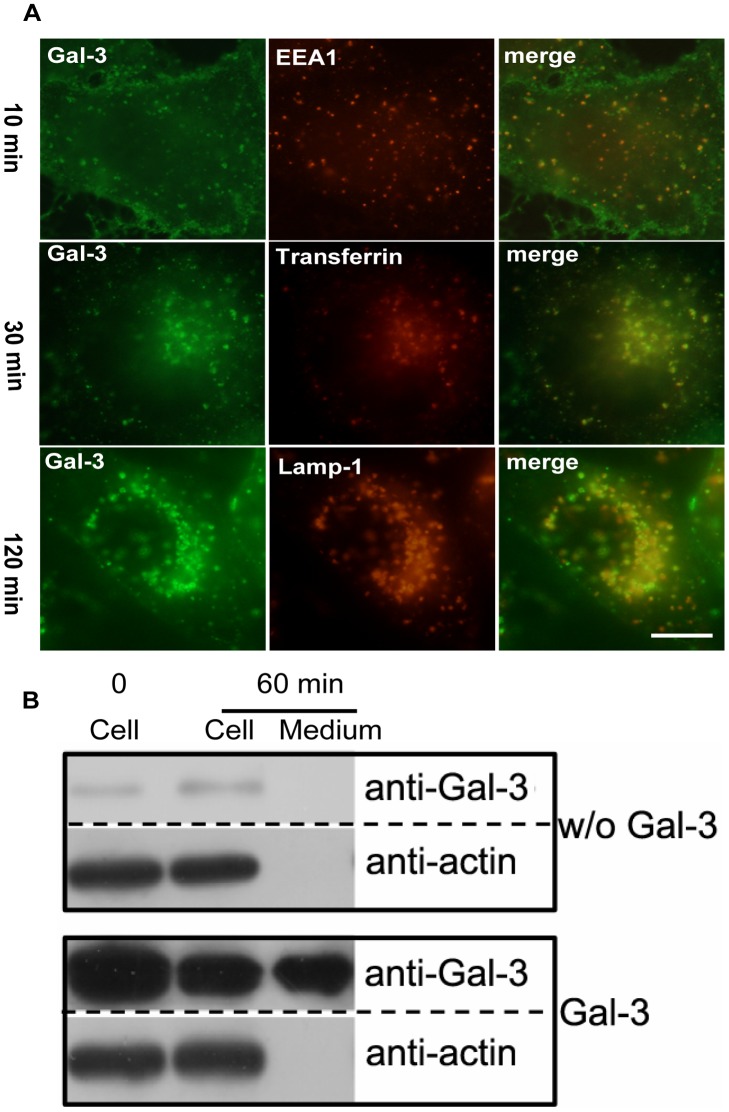
The two endocytic pathways of Gal-3 observed in HUVECs are also in HMEC-1 cells. A: Fluorescent images showing the kinetics of Gal-3 endocytosis. DTAF-Gal-3 was incubated with HMEC-1 for 10 min, 30 min, or 120 min at 37°C and then processed for IF analysis as described in Figure 4. The images in this figure were obtained with a fluorescence microscope. Scale bar, 10 µm. B:Western blot showing the exocytosis of Gal-3. The HMEC-1 cells were incubated with or without (w/o) Gal-3 at 4°C for 30 min. After extensive washing, the cells were either immediately lysed (0 min) or incubated in fresh SFM at 37°C for 60 min. After incubation, both the medium and the cells were analyzed with western blotting.

To investigate whether other vascular endothelial cells adopted the two Gal-3 transport pathways that were observed in HUVECs, Gal-3 endocytosis in HMEC-1 was studied. Morphological analysis was performed as described in Figure 4 with HMEC-1 cells that were incubated with DTAF-labeled Gal-3 at 37°C for various times. As shown in Figure 9A, Gal-3 appeared as vesicles scattered in the cytoplasm and co-localized with EEA1 at approximately 10 min. Then, it accumulated in the juxtanuclear region and co-localized with internalized transferrin at 30 min and displayed co-localization with Lamp1 at 120 min. Thus, Gal-3 was endocytosed and transported to the LE/lysosomes via EE/RE in HMEC-1.

Gal-3 exocytosis was tested with western blotting as described in Figure 7B. Gal-3 was synchronized on the cell surface and then allowed to internalize and externalize at 37°C for 60 min. As shown in Figure 9B, approximately 50% of Gal-3 was exocytosed, and 50% remained in the cells. Thus, exocytosis also occurred in HMEC-1, although the estimated percentage of exocytosed Gal-3 was different from that in HUVECs.

## Discussion

Gal-3 is intimately involved in endothelial morphogenesis and angiogenesis [14]. It has been shown that the concentration of circulating Gal-3 increases up to 5-fold in the sera of cancer patients with melanoma, breast, or gastrointestinal cancer (range, 20–950 ng/ml) compared with healthy people [21]. The concentrations of Gal-3 in the sera of colorectal cancer patients are >14-fold higher (up to 5 µg/ml) than in healthy people [28]. An increase in circulating Gal-3 has been shown to promote metastasis [22]. Here we investigated the interaction of Gal-3 with vascular endothelial cells, focusing on the endocytosis of Gal-3. To the best of our knowledge, this is the first report concerning the endocytosis of Gal-3 in endothelial cells. In this paper, we first studied the association of Gal-3 with HUVECs and found that Gal-3 at both physiological and pathological concentrations could internalized into HUVECs. We then studied cellular Gal-3 trafficking and found that it followed two pathways – either recycling or targeting to the LE/lysosomes. The co-existence of the two pathways was also observed in HMEC-1 cells. Finally, we investigated potential trafficking roles for the different structural domains of Gal-3. We found that the two endocytic pathways rely on carbohydrate recognition by the CRD and regulation by the RD but the pathways did not need the LD.

Although Gal-3 internalization into the recycling endosomes and LE/lysosomes has been observed in polarized MDCK [25] and macrophage-like cells [27], respectively, it is unknown if recycling and targeting to the LE/lysosomes occur in the same cell and how the two pathways are regulated. In this paper we not only demonstrate the co-existence of the two pathways, but also report the spatiotemporal relationships of the two pathways (Figure 10) and the roles of N- and C-terminal domains of Gal-3 in binding, endocytosis and pathway selection for the first time.

The main events that comprise the two pathways are proposed in Figure 10: (i) Gal-3 is bound via its CRD to glycol ligands on cell surface. This was demonstrated by the finding that Gal-3 was located on cell surface at 4°C and that Gal-3 association with the cells was inhibited by lactose and by the deletion of the CRD. (ii) Soon after binding, Gal-3 is endocytosed and transported to the EE/RE. This was supported by the rapid appearance of Gal-3 at the EE/RE when endocytosed at 37°C, by the rapid appearance of vesicles when temperature inhibition at 4°C was released, and by the accumulation of Gal-3 at the EE/RE when further transport was inhibited at 20°C. From the EE/RE, (iii) a fraction of Gal-3 is exocytosed, and (iv) the rest is targeted to the LE/lysosomes. This was demonstrated by kinetic studies that showed that Gal-3 accumulated in the LE/lysosomes later than EE/RE and that Gal-3 exocytosis occurred around the time of EE/RE accumulation but before LE/lysosomes accumulation. G111-250, which was mainly shuttled to along the recycling pathway, was also transported to the EE/RE, providing further evidence that the recycling pathway traverses the EE/RE. The current study does not distinguish whether each pathway involves the same or different EE/REs and whether Gal-3 could be eventually degraded in the LE/lysosomes. Our morphological studies did show that fluorescence from DTAF-Gal-3 could remain in the LE/lysosomes for over 10 h. Carbohydrate-independent endocytosis was not observed in HUVECs, which is different from that in macrophage-like cells [27].

What is the underlying mechanism that differentiates between the two trafficking pathways? One possibility is that Gal-3 binds two sets of receptors on the cell surface, with one set mediating recycling and the other targeting to the LE/lysosomes. Consistent with this hypothesis, previous studies predicted two types of receptors on HUVEC – one of high affinity (*Kd = *0.537×10^−9^) and the other of low affinity (*Kd = *7.161×10^−9^) [14]. Another possibility is that self-association or oligomerization conferred by the NT caused cross-linking of some receptors and that these cross-linked receptors acquired the ability to target to the LE/lysosomes. In line with this hypothesis, our data showed that less Gal-3 was targeted to the LE/lysosomes when the NT was partially deleted and that only a small amount of Gal-3 was targeted to LE/lysosomes when the NT was completely deleted.

Previous studies have shown that Gal3C, a CRD fragment corresponding to amino acid residues from 108 to 250, functions completely differently from the full-length protein. This truncated version fails to support neutrophil adhesion [29] and fails to induce migration and capillary tubule formation in endothelial cells in vitro and angiogenesis in vivo [7]. Conversely, it inhibited tumor growth and metastasis in the orthotopic nude mouse model of human breast cancer and also inhibited tumor growth and increased the anticancer activity of Bortezomib in a murine model of human multiple myeloma [30]. Surprisingly, our data showed that G111-250, a CRD fragment similar to Gal3C, followed transport pathways different from those of the full-length protein. Nearly all G111-250 fragments were recycled, and very few were targeted to the LE/lysosomes, unlike the 30% of full-length Gal-3 that was targeted to the LE/lysosomes. Our data provide a promising clue that targeting to the LE/lysosomes might be relevant to neutrophil adhesion, endothelia cell morphogenesis and angiogenesis, and tumor growth and metastasis.

The discovery that Gal-3 endocytosis is concomitant with binding is valuable in understanding the mechanisms of Gal-3 distribution and function. Because endocytosis would inevitably influence Gal-3 receptors by altering their distribution, residence time, turnover rate, and eventually their functions, endocytosis should be considered when interpreting experimental data. This study, however, did not eliminate the possibility that some receptors were retained on the cell surface by Gal-3, which sometimes cross-links receptors to form higher-order lattices [12], [13], [31]. Based on the higher intensity of the fluorescence in the interior of cells relative to that on the cell surface (Figure 3 & 4A), it can be estimated that any lattice-forming receptors accounted for a small proportion of Gal-3 fluorescence in the test cells. The difference between receptors involved in Gal-3 endocytosis and receptors that remained on the cell surface remains an unresolved question. At present, only a few Gal-3 receptors on HUVEC have been identified. These are integrins αvβ3 [16] and α3β1 [32], aminopeptidase N/CD13 (APN) [33], and VEGF-R2 [18]. Integrin αvβ3 appears to be the major Gal-3 binding protein in HUVEC [16]. We are currently investigating which of these is associated with endocytosis and which is specific to transport to the LE/lysosomes.

The role of Gal-3 endocytosis was previously studied in several non-endothelial cells. It has been shown that Gal-3 internalization associates with the endocytosis of β1 integrin (CD29) in breast carcinoma cells [34] and modulates adhesion plaques during cell spreading [35]. It has also been shown that Gal-3 mediates the endocytosis of advanced glycation end products and modified low-density lipoproteins in CHO cells over-expressing human Gal-3 [36]. The discovery that Gal-3 takes different endocytic pathways in different macrophage-like cell types suggests that Gal-3 endocytosis might relate to particular immunological responses [26].

Due to the high expression level of Gal-3 in tumor cells and in the blood stream, the endothelial cells near tumor tissues and in the blood vessels inevitably encounter high concentrations of Gal-3. It is conceivable that the endocytosis of a large quantity of Gal-3 would have significant consequences on cell properties. Further investigation into the effects of Gal-3 endocytosis on cellular processes would help elucidate the mechanism behind Gal-3 induced angiogenesis and metastatic cell adhesion to the endothelium.

## Materials and Methods

### Materials

Lactose-Sephorose CL-6B was prepared using lactose and Sepharose CL-6B according to the previously published protocols [37]. Monoclonal antibodies against Gal-3 (A3A12) and Lamp-1 (H4A3) were purchased from Santa Cruz Biotechnology, Inc. The monoclonal antibody against EEA1 was obtained from Becton, Dickinson and Company. The polyclonal antibody against Golgin-97 was previously described [38]. Transferrin, biotin amidocaproate NHS ester and the polyclonal antibody against α-actin were purchased from Sigma Aldrich. The rhodamine-streptavidin used for detecting biotin was purchased from Boster Bio-engineering limited company, Wuhan. All the secondary antibodies labeled with fluorescein or HRP were purchased from Zhongshan Jinqiao Biotechnology Co., Ltd. The dye 5-(4, 6-dichlorotriazinyl) aminofluorescein (5-DTAF) was purchased from Invitrogen. The dye Cy3 was purchased from GE Healthcare (cat. no. PA23001).

### Cell Culture

The HUVECs were purchased from American Type Culture Collections and cultured in F-12K medium supplemented with 10% heat-inactivated fetal bovine serum (GIBCO) with 0.1 mg/ml heparin and 0.03 mg/ml endothelial cell growth supplement (ECGS). The HMEC-1 cells were a gift from professor Kan Ding (Glycochemistry and Glycobiology Laboratory, Shanghai Institute of Materia Medica, Chinese Academy of Sciences) who obtained the cell lines from the Centers for Disease Control and Prevention (Atlanta, GA) [39]. HMEC-1 were cultured in MCDB131 medium supplemented with 15% fetal bovine serum with 10 ng/ml EGF and 2 mM L-Glutamine All the cultures were maintained at 37°C in a humidified atmosphere of 95% air and 5% CO_2_.

### Preparation of Recombinant Human Gal-3, GST-Gal-3 and Truncated Gal-3

Plasmid pOTBT (Wuhan Sanying Biotechnology, Inc.) containing the full sequence of human Gal-3 was used as a PCR template to amplify the cDNAs of various forms of Gal-3. The forward and reverse primers that were used are listed in Table 1. The expression plasmids for Gal-3, G13-250, G69-250 and G111-250, which contained amino acid 1 to 250 (i.e. the full length of Gal-3), 13 to 250, 69 to 250, and 111 to 250 respectively, were constructed by inserting the appropriate cDNAs into the vector pET-22b (+) between the *NdeI* and *BamHI* cut sites and confirmed by DNA sequencing. *Escherichia coli BL21 (DE3)* cells were transformed with these constructs and induced to express proteins by incubating with 0.2 mM IPTG for 12 h at 25°C. The proteins were extracted and purified with lactose-Sepharose CL-6B according to previously reported protocols [37]. The expression plasmids of fusion protein GST-Gal-3 and GST-G1-108, which contained amino acid 1 to 250 and 1 to 108, respectively, were constructed by inserting cDNAs into the vector pGEX-4T-2 between *BamHI* and *EcoRI* cut sites. The fusion proteins were expressed in *Escherichia coli DH5α* and induced as described above. They were purified by glutathione affinity chromatography according to the manufacturer’s instructions. The purity of the proteins was detected using SDS-PAGE (Figure 1B).

**Table 1:** Primers.

Proteins	Forward primer	Reverse primer
Gal-3	agccatatggcagacaatttttc	tgggatccagattatatcatggtat
G13-250	taacatatggggtctggaaaccaa	tgggatccagattatatcatggtat
G69-250	taacatatggcttatcccggagca	tgggatccagattatatcatggtat
G111-250	ttacatatggctgggccactgatt	tgggatccagattatatcatggtat
GST-Gal-3	ataggatccgcagacaatttttcgct	ggcgaattcagattatatcatgg
GST-G1-108	ataggatccgcagacaatttttcgct	tagaattcttagccataggggcca

### Preparation of Polyclonal Antibody Against Gal-3

Recombinant human Gal-3 (0.75 mg) in 50% Freund’s complete adjuvant was injected subcutaneously into a rabbit followed by two booster injections that each consisted of 0.5 mg of Gal-3 in 50% Freund’s incomplete adjuvant. Serum was collected 10 days after the final immunization and evaluated using ELISA. Serum specificity was tested using western blotting (Figure 8A).

### Biotinylation and Fluorescent Labeling of Proteins

For biotinylation, Gal-3 (2 mg/ml in PBS, pH 7.4) was mixed with biotin amidocaproate NHS ester (20 mg/ml in DMSO) at 1∶15 mole/mole and incubated for 30 to 60 min at room temperature, according to the manufacturer’s instructions. The biotinylated Gal-3 was then purified by extensive dialysis against PBS. For Cy3 labeling, 2 mg/ml of transferrin in 1 M sodium carbonate-sodium bicarbonate buffer, pH 9.0, was incubated with the dye at room temperature for 1 h according to the manufacturer’s instructions. The labeled proteins were separated from remaining free dye by extensive dialysis in PBS. For DTAF labeling, 5-DTAF (10 mg/ml in DMSO) was added to various Gal-3 solutions (Gal-3, G13-250, G69-250, G111-250, GST-Gal-3, GST-G1-108, at ≥2 mg/ml in 1 M bicarbonate buffer, pH 9.0) to a final concentration of 1 mg/ml. The reactions were incubated for 1 h at room temperature and then terminated by incubation with 1 M glycine (dissolved in PBS) for another 1 h. The labeled proteins were separated from remaining free dye by extensive dialysis in PBS.

### Association Assays

The cells cultured on 6-, 12- or 24-well plates for 2 days were washed twice with serum-free medium (SFM) and then incubated with various forms of Gal-3 in SFM at 4, 20 or 37°C for various times as indicated. In some cases, 100 mM lactose or sucrose was included together with Gal-3. After incubation, the cells were placed on ice and washed five times with cold SFM. Cell-associated Gal-3 was analyzed with western blot.

### Exocytosis Assays

The cells were incubated with 15 µg/ml of Gal-3 in SFM at 4 or 37°C for 30 min to allow Gal-3 binding or endocytosis. Afterwards, the cells were placed on ice, washed five times with cold SFM, and then incubated in fresh SFM without Gal-3 at 37°C for various times. The media were collected and examined for Gal-3 using western blotting. In some experiments, both the cells and the media were examined.

### SDS-PAGE and Western Blotting Assays

For the detection of cell-associated Gal-3, the cells were detached from plates using a cell scraper in lysis buffer (50 mM Tris/acetate, pH 7.4, 0.5% Triton X-100, 150 mM sodium chloride, 0.1 mM PMSF and Roche complete protease inhibitor cocktail) and incubated on ice for 60 min. The cell lysate was centrifuged at 13,000 g for 15 min at 4°C. The supernatant was collected and mixed with loading sample buffer. For detection of Gal-3 in the media, the proteins in the media were precipitated by TCA and then dissolved in loading sample buffer. Proteins from both cells and media were separated using a 12% acrylamide gel for SDS–PAGE and transferred to PVDF membranes. The membranes were blocked with 5% milk in PBST (PBS containing 0.1% Tween 20) overnight at 4°C. They were then incubated for 1 h with primary antibodies (A3A12 at 1∶1000, Gal-3 antisera at 1∶5000, anti-α-actin at 1∶5000). After three washes with PBST, the membranes were incubated with HRP-conjugated secondary antibodies for 1 h. All protein blots were developed using an ECL Plus Western Blotting Detection Kit from GE Healthcare.

### Immunofluorescence Assays (IF)

The cells were planted and grown on 12-mm round glass cover slips at 5×10^4^ cells/well. Forty hours later, the cells were washed with SFM and then incubated with either biotinylated or DTAF-labeled Gal-3 or DTAF-labeled Gal-3 together with Cy3-labeled transferrin at 4, 20 or 37°C for various times as indicated. The cells were placed on ice, washed five times with cold PBSCM (PBS containing 1 mM Ca^2+^ and 1 mM Mg^2+^) and fixed in 3% paraformaldehyde followed by permeabilization in 0.1% Triton X-100. The cells were then incubated with primary antibodies against EEA1 at a 1∶30 dilution, Lamp 1 at 1∶100 dilution, or golgin-97 at 1∶100 dilution. After incubation with appropriate secondary antibodies, the cells were mounted and viewed under a fluorescence microscope (Olympus BX51) or a confocal microscope (Olympus BX81) with a 60X lens.

## Supporting Information

Figure S1 **Comparison of full-length and truncated Gal-3.** Identical concentrations of DTAF-Gal-3, DTAF-G13-250, DTAF-G69-250 and DTAF-G111-250 were incubated with HUVEC at 37°C for 120 min and then processed for IF analysis. A: Comparison of fluorescence intensities. B: Co-localization with Lamp-1.
(TIF)
