# Supplementary figures and images for: The Two Endocytic Pathways Mediated by the Carbohydrate Recognition Domain and Regulated by the Collagen-like Domain of Galectin-3 in Vascular Endothelial Cells
Source: PLoS One. 2012 Dec 26;7(12):e52430. doi: 10.1371/journal.pone.0052430 (PMC3530513; doi:10.1371/journal.pone.0052430)

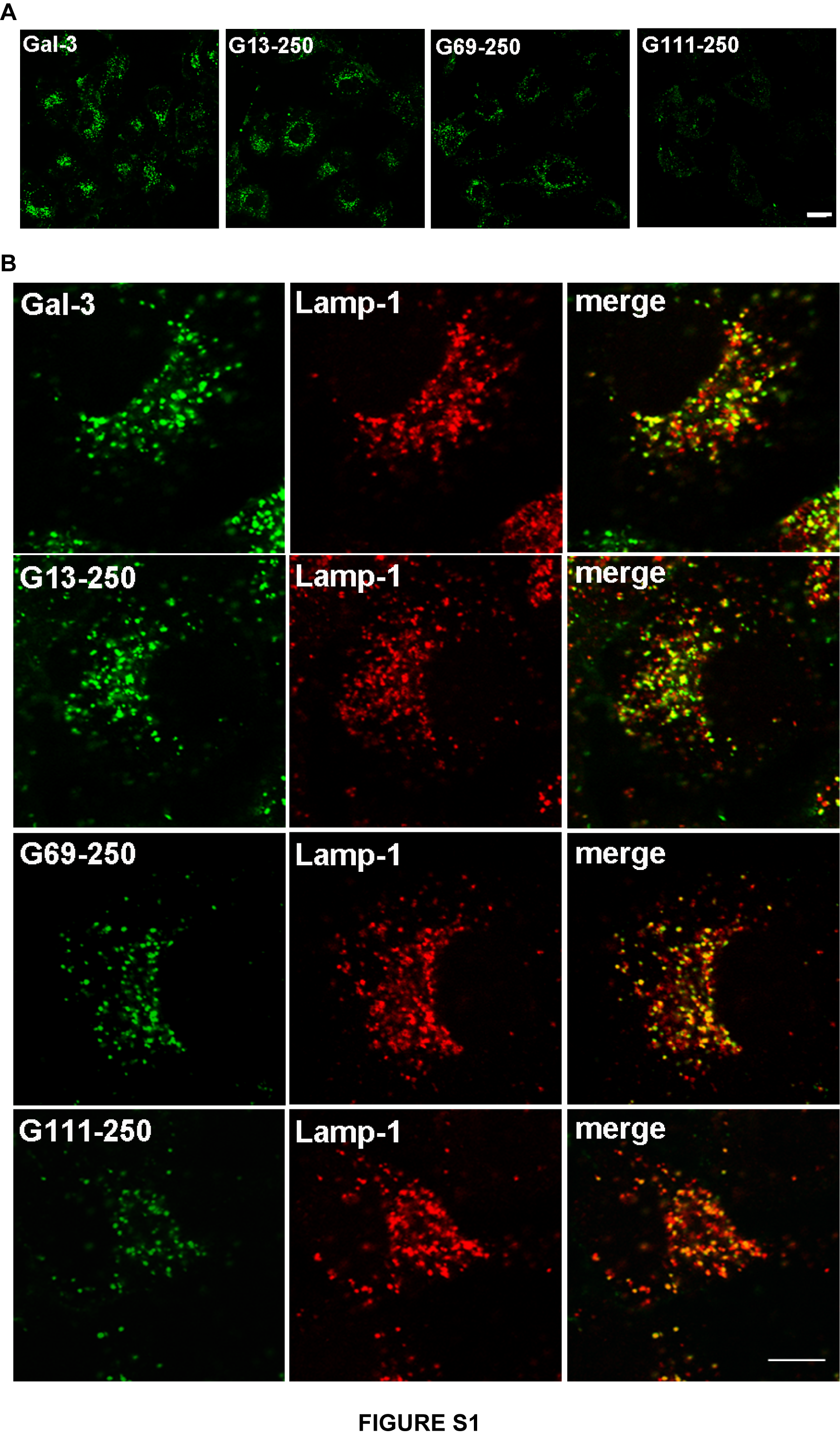

Supplement: Figure S1 — Comparison of full-length and truncated Gal-3. Identical concentrations of DTAF-Gal-3, DTAF-G13-250, DTAF-G69-250 and DTAF-G111-250 were incubated with HUVEC at 37°C for 120 min and then processed for IF analysis. A: Comparison of fluorescence intensities. B: Co-localization with Lamp-1. (TIF) [file pone.0052430.s001.tif]
